# THE UNIQUE IMPORTANCE OF RELATIONSHIP QUALITY FOR COMMUNITY-DWELLING OLDER ADULTS

**DOI:** 10.1093/geroni/igz038.3490

**Published:** 2019-11-08

**Authors:** Ashley M Tate, Lynn Martire

**Affiliations:** The Pennsylvania State University, University Park, Pennsylvania, United States

## Abstract

Social networks can directly influence the health and well-being of older adults. Some work has suggested that network growth is associated with increased well-being. However, little is known about how the quality of relationships with confidants may be associated with better psychological well-being over and above the number of confidants. We aimed to test the hypothesis that feeling closer to confidants would be associated with lower anxiety and fewer depressive symptoms above and beyond the influence of the number of confidants as well as the number of children and grandchildren. To test this hypothesis, we collected data during face-to-face interviews with 131 community-dwelling adults who were between the ages of 58 and 94. Participants’ gender, age, marital status, self-rated health, and cognitive function were included as covariates in the models. In line with predictions, regression analyses showed that average closeness with confidants predicted significantly lower reports of anxiety (p < .05) and depressive symptoms (p < .001). Additionally, the number of confidants was not significantly associated with anxiety or depressive symptoms. Interestingly, having a greater number of children and grandchildren was associated with increased anxiety symptoms. These results extend previous work by suggesting that the quality of the relationship with confidants is more important for psychological well-being than the number of confidants. Future work should test these associations longitudinally so that directionality can be inferred.

